# Evaluation of Proinflammatory, NF-kappaB Dependent Cytokines: IL-1α, IL-6, IL-8, and TNF-α in Tissue Specimens and Saliva of Patients with Oral Squamous Cell Carcinoma and Oral Potentially Malignant Disorders

**DOI:** 10.3390/jcm9030867

**Published:** 2020-03-21

**Authors:** Karolina Babiuch, Beata Kuśnierz-Cabala, Barbara Kęsek, Krzysztof Okoń, Dagmara Darczuk, Maria Chomyszyn-Gajewska

**Affiliations:** 1 Jagiellonian University Medical College, Faculty of Medicine, Institute of Dentistry, Chair and Department of Periodontology and Clinical Oral Pathology, Montelupich 4, 31-155 Krakow, Poland; barbara.kesek@uj.edu.pl (B.K.); dagmara.darczuk@uj.edu.pl (D.D.); mdgajews@cyf-kr.edu.pl (M.C.-G.); 2 Jagiellonian University Medical College, Faculty of Medicine, Chair of Clinical Biochemistry, Department of Diagnostics, Kopernika 15A, 31-501 Krakow, Poland; mbkusnie@cyf-kr.edu.pl; 3 Jagiellonian University Medical College, Faculty of Medicine, Chair of Pathomorphology, Grzegórzecka 16, 31-531 Krakow, Poland; k.okon@uj.edu.pl

**Keywords:** oral squamous cell carcinoma, oral potentially malignant disorders, inflammation, biomarkers, cytokines

## Abstract

Background: Oral squamous cell carcinoma (OSCC) is a life-threatening disease. It could be preceded by oral potentially malignant disorders (OPMDs). It was confirmed that chronic inflammation can promote carcinogenesis. Cytokines play a crucial role in this process. The aim of the study was to evaluate interleukin-1alpha (IL-1α), interleukin-6 (IL-6), interleukin-8 (IL-8), and tumor necrosis factor alpha (TNF-α) in tissue specimens and saliva of patients with OSCC and OPMDs. Methods: Cytokines were evaluated in 60 tissue specimens of pathological lesions (OSCCs or OPMDs) and in 7 controls (normal oral mucosa, NOM) by immunohistochemistry and in saliva of 45 patients with OSCC or OPMDs and 9 controls (healthy volunteers) by enzyme-linked immunosorbent assays. Results: Immunohistochemical analysis revealed significantly higher expression of IL-8 in OSCC specimens and TNF-α in OSCCs and OPMDs with dysplasia as compared to NOM. Moreover, expression of TNF-α was significantly higher in oral leukoplakia and oral lichen planus without dysplasia, whereas expression of IL-8 only in oral leukoplakia without dysplasia in comparison with NOM. Salivary concentrations of all evaluated cytokines were significantly higher in patients with OSCC than in controls. Moreover, levels of IL-8 were significantly higher in saliva of patients with OPMDs with dysplasia as compared to controls and in OSCC patients as compared to patients with dysplastic lesions. There was also significant increase in salivary concentrations of IL-6, IL-8 and TNF-α in patients with OSCC as compared to patients with OPMDs without dysplasia. Conclusion: The study confirmed that proinflammatory, NF-kappaB dependent cytokines are involved in pathogenesis of OPMDs and OSCC. The most important biomarker of malignant transformation process within oral mucosa among all assessed cytokines seems to be IL-8. Further studies on a larger sample size are needed to corroborate these results.

## 1. Introduction

Oral cancer is quite common disease and has an increasing worldwide trend [1]. Histologically, over 90% of malignancies affecting this region are diagnosed as oral squamous cell carcinoma (OSCC) [2]. OSCC is responsible for approximately 4% of all malignancies [3]. It has invasive behavior and high risk of metastases. The mortality rate associated to OSCC is high and has remained unchanged over the past decades [4]. The main reason is too late diagnosis.

There are some clinically defined precursor lesions, such as oral erythroplakia, oral leukoplakia, oral submucous fibrosis, and oral lichen planus, that can precede cancer development within the oral mucosa. All these lesions should be called oral potentially malignant disorders (OPMDs) [5]. The term was recommended in year 2005 during one of the WHO workshops [6].

The identification of OPMDs with higher risk of malignant transformation and OSCCs at the early stage of development seems to be a matter of great importance and the best way to improve OSCC statistics.

Oral carcinogenesis is a complex process in which genetic events result in successive molecular changes that lead to the disruption of cell proliferation, growth, and differentiation [7]. The kinetics of this event is a result of the interactions between tumor and host, especially the immune system [8]. The role of inflammation in carcinogenesis was suggested for the first time by Rudolf Virchow more than 150 years ago [9]. Various studies have confirmed that chronic inflammation can influence cell homeostasis and various metabolic processes, inducing changes at the genomic level, which can promote carcinogenesis [10]. Inflammation stimulates the activation of cytotoxic mediators, such as reactive oxygen species (ROS) and reactive nitrogen species (RNS), which play a major role in DNA damages. DNA damage accumulation is responsible for the initiation of carcinogenesis through the enhancement of genomic instabilities. Moreover, several inflammatory factors can facilitate the migration and invasion of neoplastic cells, leading to cancer progression [11].

Cytokines are critical regulators of tumor microenvironment and chronic pro-tumorigenic inflammation [12]. They are soluble, low molecular weight, multifunctional polypeptides that are produced mainly by cells of the innate and adaptive immune system but also by resident tissue and tumor cells [8]. They influence many aspects of cellular behaviors, such as growth, differentiation, and function. Their physiological activities are dysregulated during inflammation and carcinogenesis. The studies confirmed the crucial role of proinflammatory cytokines in carcinogenesis process, including the development of lung cancer [13,14], hepatocarcinoma [15], colorectal cancer [16], as well as OSCC [17].

The transcription factor, nuclear factor-kappaB (NF-kB) is an early response gene promoting the expression of a series of cytokines with proinflammatory, proangiogenic, and immunoregulatory activity which play an important role in carcinogenesis. Aberrant NF-kB regulation has been observed in many cancers [18,19]. Studies have demonstrated the activation of NF-kB in OSCC and elevated expression of its downstream proinflammatory cytokines in tissues, serum, tissue infiltrating lymphocytes (TIL) and cell lines of OSCC, including interleukin-1alpha (IL-1α), interleukin-6 (IL-6), interleukin-8 (IL-8), and tumor necrosis factor alpha (TNF-α) [20].

IL-1α modulates various growth-promoting pathways, including anti-apoptotic signaling and cellular proliferation [21]. It was also observed that IL-1α released from OSCC cells stimulates carcinoma-associated fibroblasts (CAFs) to secrete CCL7, CXCL1, and IL-8, thereby facilitating cancer invasion [22].

IL-6 is a multifunctional cytokine with growth-promoting and anti-apoptotic activity [18,23]. There is evidence that IL-6 regulates activation of the Janus kinases (JAK) and signal transducers and activators of transcription (STATs), which then stimulate pathways involving mitogen-activated protein kinase (MAPK), which in turn supports cancer development [24].

IL-8, a member of the chemokine family, acts on two receptors, namely CRCX-1 and CRCX-2, that are located on tumor-associated macrophages, neutrophils, and cancer cells. Their presence on cancer cells strongly suggests that IL-8 is an important chemokine for cancer cells environment. The carcinogenic potential of IL-8 results from its ability to neutrophil recruitment, angiogenic potential, proliferation and survival promotion, as well as protection from apoptosis [24].

TNF-α is a pleiotropic cytokine. It is known that the TNF-TNF receptor system plays an important role in inflammation, angiogenesis, programmed cell death, and proliferation, which are all crucial components in malignant transformation process [25]. It was also discovered that TNF-α can directly damage DNA of cells and lead to their malignant transformation through induction of reactive oxygen species (ROS) [26]. Additionally, TNF family members contribute to immune suppression [18].

There are some immunohistochemical studies that confirmed the role of pro-inflammatory, NF-kB dependent cytokines in malignant transformation process within oral mucosa, however evidence is rather scarce. It was revealed that IL-6 and TNF-α can promote malignant transformation in patients with oral submucous fibrosis [27] and with oral lichen planus [28]. Moreover, it was reported that the expression of TNF-α is significantly increased in lesions exhibiting epithelial dysplasia [29].

In recent years, the role of saliva for early detection of oral cancer has been intensively studied [30,31,32,33]. Due to the fact that saliva can be collected in an easy and noninvasive way, it seems to be a very attractive diagnostic material [34]. Proinflammatory cytokines have also been investigated in saliva as potential biomarkers of OPMDs and OSCC, and the current results are encouraging [24,35,36,37].

The aim of the presented study was to evaluate IL-1α, IL-6, IL-8, and TNF-α in tissue specimens and saliva of patients with oral squamous cell carcinoma and oral potentially malignant disorders such as oral leukoplakia and oral lichen planus to confirm the potential of proinflammatory, NF-kappaB dependent cytokines as biomarkers of malignant transformation process within the oral mucosa.

## 2. Materials and Methods

### 2.1. Study Group

Sixty patients with diagnosis of OSCC or OPMDs such as oral leukoplakia and oral lichen planus were included into the study. They were diagnosed in the Chair of Periodontology and Clinical Oral Pathology and Department of Oral Surgery, Institute of Dentistry, Jagiellonian University Medical College in Krakow between 2011 and 2015. The diagnosis was made on the basis of clinical and histopathological examination using the WHO criteria [38]. The approval of the Bioethics Committee of the Jagiellonian University (KBET/290/B/2011, KBET/122.6120.183.2015) and the informed consent of the patients were obtained before collection of saliva and evaluation of tissue specimens. The study was performed in accordance with the Helsinki Declaration of 2008.

### 2.2. Histopathology and Immunohistochemistry

The formalin-fixed, paraffin-embedded blocks of 60 tissue samples collected from patients of the study group were sectioned (2 µm). Normal oral mucosa (NOM) in margins of 7 formalin-fixed, paraffin-embedded archival blocks of fibromas were used as controls. For histopathological examination, the sections were stained with hematoxylin and eosin (H&E). OSCCs were graded as well, moderately, and poorly differentiated using the standard WHO criteria [39]. The criterion for judging the malignant potential of OPMDs is mainly the presence and degree of dysplasia [39]. OPMDs were classified histologically into stages with increasing risk of developing into OSCC, namely as mild, moderate, and severe epithelial dysplasia according to the WHO criteria [6,40].

For immunohistochemistry, the sections were deparaffinized and rehydrated. After antigen retrieval, slides were incubated with antibodies: Anti-IL-1α rabbit polyclonal IgG (30 min., room temp.) and Anti-IL-6 mouse monoclonal IgG (30 min., room temp.) purchased from Santa Cruz Biotechnology Inc. (Dallas, TX, USA) and Anti-IL-8 mouse monoclonal IgG (60 min., room temp.) and Anti-TNF-α rabbit polyclonal IgG (60 min., room temp.) purchased from Abcam Plc. (Cambridge, UK) diluted (1:50) in Tris-buffer solution (TBS) followed by incubation with Large Volume HRP Polymer (Lab Vision Corporation, Fremont, CA, USA). For visualization of the antigen 3,3-diaminobenzidine tetrahydrochloride (Lab Vision Corporation, Fremont, CA, USA) was added. Slides were counterstained with hematoxylin (Thermo Fisher Scientific, Waltham, MA, USA).

Taking into account that epithelium and stroma contain completely different cells, we analyzed them separately. Depend on the proportion of the positively stained cells and intensity of staining, a semiquantitative immunoreactive score from 0 to 6 was calculated separately for epithelial/cancer cells and stromal cells (Table 1). The overall score was not calculated. The score was elaborated by authors based on the literature [41,42,43].

### 2.3. Laboratory Tests

Whole unstimulated saliva (WUS) was collected from 45 subjects of the study group: 9 patients with OSCC, 7 with oral epithelial dysplasia (OED), 16 with oral leukoplakia without dysplasia (OL), and 13 with oral lichen planus without dysplasia (OLP). Individuals with a history of any systemic inflammatory disease, individuals suffering from inflammatory conditions in the oral cavity (e.g., dental abscess, pericoronitis, gingivitis, periodontitis), patients treated because of OSCC in the past, individuals taking drugs that induced hyposalivation (e.g., anticholinergics, antihistamines, antihypertensives, and beta adrenal blockers), and individuals using secretagogues were excluded from this part of the study. None of the lesions had been treated in any manner prior to sample collection. Samples of WUS of nine volunteers without any systemic diseases and without any pathological lesion within oral mucosa were used as controls.

WUS samples were collected between 9.00 and 11.00 a.m. The subjects were instructed to refrain from eating, drinking, using chewing gum, and smoking for at least 90 min before collection of saliva. Samples were obtained by requesting the subjects to swallow first, tilt their head forwards, and expectorate the saliva into plastic vials for 10 min [44]. Samples were stored at −80 °C and centrifuged at 6000 rpm for 20 min to remove squamous cells and debris before the biochemical analysis.

Levels of particular cytokines in saliva samples were measured using commercially available enzyme-linked immunosorbent assays: Human IL-1α/IL-1F1 Quantikine ELISA kit, Human IL-8/CXCL8 Quantikine ELISA kit and Human TNF-α Quantikine HS ELISA kit purchased from R&D Systems (Minneapolis, MN, USA), and Human IL-6 High Sensitivity ELISA kit purchased from Gen-Probe Diaclone SAS (Besançon, France). The measurements were taken with an Automatic Micro ELISA Reader ELX 808 (BioTek Instruments Inc., Winooski, VT, USA). The minimum detectable dose (MDD) of IL-1α was less than 1.0 pg/mL, for hsTNF-α ranged from 0.038–0.191 pg/mL, for IL-8 ranged from 1.5–7.5 pg/mL, and for hsIL-6 was 0.81 pg/mL.

All laboratory tests were conducted in the Diagnostic Department, Chair of Clinical Biochemistry, Jagiellonian University Medical College, Krakow, Poland.

### 2.4. Statistical Analysis

For categorical variables, frequency and percentage were calculated. For continuous variables, minimum (Min), maximum (Max), median (Me) and interquartile range (IQR) were calculated. For qualitative data, differences between groups were analyzed by Fisher’s exact test. For quantitative data, differences between groups were analyzed by Kruskal-Wallis test and post-hoc analyses were performed with Dunn’s test. *p*-values less than 0.05 were considered significant. Analyses were performed using the Statistical Package for Social Sciences (SPSS, version 19.0) and the R Project for Statistical Computing (www.R-project.org).

## 3. Results

### 3.1. Histopathology and Immunohistochemistry

On the basis of histopathological examination and clinical data, 14 tissue samples were diagnosed as OSCC, 21 as oral leukoplakia (hyper- and/or parakeratosis) without dysplasia (OL), 15 as oral lichen planus without dysplasia (OLP), and 10 as OED (5 cases as hyper- and/or parakeratosis with dysplasia and 5 cases as lichenoid dysplasia). Using the standard WHO criteria, 6 cases of OSCC were classified as well differentiated, another 6 cases as moderately and 2 cases as poorly differentiated. Among OED cases all but only one were classified as mild and one as severe epithelial dysplasia according to the WHO criteria.

Immunohistochemical staining revealed differences in distribution of particular cytokines within the epithelium between different types of lesions (Table 2). IL-1α was present more often within all layers of the epithelium in OSCCs than in OPMDs and it was present within all layers of the epithelium in none of the specimens assessed as normal oral mucosa. In turn, TNF-α was present within all layers of the epithelium in almost all cases of OED and OSCC specimens and only in one third cases of specimens assessed as NOM. Moreover, TNF-α was not present in any layer of the epithelium of almost one third of NOM specimens. When only OSCC, OED, and NOM cases were included in the statistical analysis, significant differences in distribution of IL-8 within the epithelium between compared lesions were confirmed. IL-8 was present within all layers of the epithelium in almost 65% of OSCC cases and in none layer of the epithelium of all specimens assessed as NOM.

Analysis of immunoreactive scores confirmed significant differences in immunoreactivity for IL-8 and TNF-α (Table 3 and Table 4). When only OSCC, OED, and NOM cases were compared, immunoreactivity for IL-8 was significantly higher in epithelial/cancer cells and in stroma of OSCCs in comparison with NOM specimens (*p* = 0.0073 and 0.032, respectively), whereas immunoreactivity for TNF-α was markedly higher in epithelium and stroma of OEDs in comparison with NOM cases (*p* = 0.019 and 0.0038, respectively) and in epithelium/cancer cells of OSCCs as compared to NOM specimens (*p* = 0.011). Moreover, immunoreactivity for TNF-α was significantly higher in stroma of OED cases than in OSCCs (*p* = 0.0102). When all types of specimens were included into statistical analysis, significant differences in immunoreactivity for IL-8 in stroma and for TNF-α in epithelium and stroma between oral leukoplakia without dysplasia and NOM cases (*p* = 0.022, *p* = 0.0017, and 0.047, respectively) as well as for TNF-α in epithelium between oral lichen planus without dysplasia and NOM specimens (*p* = 0.0071) were also revealed.

Below we present photos of immunohistochemical staining for IL-1α, IL-8, and TNF-α in selected specimens of OSCCs and OPMDs (Figure 1, Figure 2, Figure 3, Figure 4, Figure 5, Figure 6, Figure 7 and Figure 8).

### 3.2. Laboratory Tests

Subjects characteristics are given in Table 5. There were no significant differences in age, sex, as well as cigarette use and alcohol consumption between compared groups.

Statistical analysis revealed significant differences in levels of all measured cytokines when only patients with OSCC, OED, and healthy volunteers were compared. Concentrations of IL-1α, IL-6, IL-8, and TNF-α were markedly higher in saliva of patients with OSCC in comparison with healthy volunteers (*p* = 0.017, 0.0012, 0.0001, and 0.0012, respectively). Moreover, levels of IL-8 were significantly higher in saliva of patients with OED as compared to controls (*p* = 0.0492) and in OSCC patients as compared to patients with OED (*p* = 0.0345).

**Figure 9:**
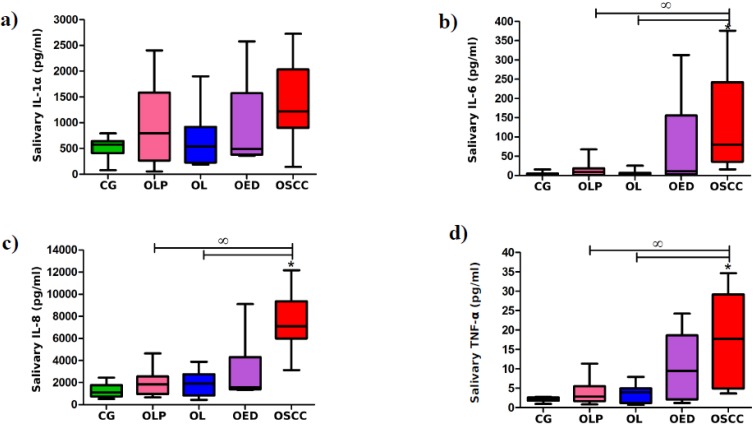
Salivary levels of IL-1α (**a**), IL-6 (**b**), IL-8 (**c**), and TNF-α (**d**) in control group (CG) and patients with oral lichen planus without dysplasia (OLP), oral leukoplakia without dysplasia (OL), oral epithelial dysplasia (OED), or oral squamous cell carcinoma (OSCC); the median and interquartile range (box), and percentile 5–95% range (whiskers) are shown; * means significant difference vs. CG; ꝏ means significant difference vs. OSCC (*p* < 0.05).

However, when all groups were analyzed, only levels of IL-6, IL-8, and TNF-α were markedly higher in patients with OSCC as compared to controls (*p* = 0.0041, 0.0004, and 0.0041, respectively). Concentrations of IL-6, IL-8, and TNF-α were also markedly higher in OSCC group as compared to subjects with oral leukoplakia without dysplasia (*p* = 0.0012, 0.0000, and 0.0492, respectively) and oral lichen planus without dysplasia (*p* = 0.0084, 0.0002, and 0.0212, respectively) (Figure 9).

## 4. Discussion

Alterations in host immunity, inflammation, angiogenesis, and metabolism have been noted as the prominent pathological features in patients with oral cancer [45]. NF-kappaB dependent cytokines are molecular messengers highly involved in all these processes [24]. Altered levels of proinflammatory, NF-kappaB dependent cytokines have been reported not only in patients with OSCC but also in patients with OPMDs, such as oral leukoplakia, oral lichen planus, and OSF [46]. There are numerous studies in which levels of proinflammatory cytokines were assessed in body fluids of patients with OSCC or OPMDs, however, in most of them only one cytokine and one type of OPMDs was considered. Moreover, in some of the previous studies exclusion criteria were not restrictive. In turn, the evidence on the expression of proinflammatory, NF-kappaB dependent cytokines in tissue samples of OSCCs and OPMDs is very limited, especially in OPMDs. Thus, the present study is unique. We decided to evaluate the panel of four proinflammatory, NF-kappaB dependent cytokines (IL-1α, IL-6, IL-8, and TNF-α) not only in saliva, but also in tissue specimens of OSCCs and OPMDs such as oral leukoplakia and oral lichen planus. We compared the expression of IL-1α, IL-6, IL-8, and TNF-α in epithelial and stromal cells between different types of tissue specimens implementing the immunoreactive score. To the best of our knowledge, this is the first study designed in this way. Moreover, to reduce the risk of interfering variables affecting salivary concentrations of assessed cytokines we implemented strict exclusion criteria. Subjects with acute or chronic inflammatory conditions in the oral cavity, such as dental abscess, pericoronitis, gingivitis, or periodontitis, patients with systemic inflammatory diseases and patients taking medications that can alter salivary flow were not included into the salivary analysis. All analyzed groups were also comparable in terms of age, gender, cigarette smoking and alcohol drinking. It should be also underline that this is the first such study carried out in the Polish population.

The results of immunohistochemical staining confirmed the expression of IL-1α, IL-6, IL-8, and TNF-α in OSCCs. Analyzed cytokines were observed within epithelial/cancer cells of most OSCC cases and in stroma of all OSCC tissue specimens. Likewise, Woods et al. confirmed intracellular production of IL-1 and IL-6 in all analyzed invasive OSCCs, whereas Chen et al. detected IL-1α, IL-6, and IL-8 within keratin-positive malignant epithelium of all analyzed OSCCs in situ [47,48]. In turn, de Oliveira et al. revealed presence of IL-6 and IL-8 in inflammatory cells in invasive front of all analyzed OSCCs [8]. These results showed that proinflammatory, NF-kappaB dependent cytokines, which regulate innate and adaptive immune response, are produced not only by inflammatory cells in the tumor microenvironment, but also by tumor cells. Expression of these proinflammatory and proangiogenic cytokines in OSCCs indicate that they may play a role in the increased pathogenicity of OSCC by providing a growth advantage.

The present study also confirmed the expression of IL-1α, IL-6, IL-8, and TNF-α in oral leukoplakia and oral lichen planus specimens. All assessed cytokines were observed in stroma of every OPMD and in epithelial cells of most analyzed cases. However, IL-8 was present in the smallest number of OPMDs samples as compared to other assessed cytokines. Haque et al. confirmed the expression of IL-1α and IL-6 in stroma and in epithelial cells of specimens of oral submucous fibrosis, whereas Sclavounou et al. reported the expression of TNF-α in epithelial cells and proinflammatory cells of most analyzed oral lichen planus specimens [49,50]. These results indicate that proinflammatory cytokines, especially IL-1α, IL-6, and TNF-α could play an important role in the pathogenesis of OPMDs.

The comparison of immunoreactivity for particular cytokines between different types of tissue specimens analyzed in this study revealed that the expression of IL-8 and TNF-α was markedly increased in OSCCs in comparison with tissue specimens assessed as normal oral mucosa, whereas in OED specimens the expression of TNF-α was notably altered. These results together with the fact that IL-8 was not present in epithelial cells of specimens assessed as normal oral mucosa, whereas it was present within all layers of the epithelium in most cases of OSCCs indicate that IL-8 and TNF-α could play a leading role among proinflammatory, NF-kappaB dependent cytokines in malignant transformation process within the oral mucosa.

Because of too small samples and a large disproportion in numbers between subgroups with different grade of OED and with different differentiation grade of OSCC, it was not possible to check whether the grade of OED (mild, moderate, and severe) and the differentiation grade of OSCC (well, moderate, and poor) significantly influence the expression of cytokines.

Analysis of saliva revealed markedly higher levels of IL-1α, IL-6, IL-8, and TNF-α in OSCC patients in comparison with healthy individuals when only OSCC, OED, and controls were taken into consideration, whereas only IL-6, IL-8, and TNF-α when all groups were analyzed. These results are in line with other studies. Rajkumar et al. and Lee at al. reported significantly higher salivary levels of IL-6, IL-8, and TNF-α in OSCC patients in comparison with controls [51,52], whereas Rhodus et al. observed significantly higher levels of IL-1α, IL-6, IL-8, and TNF-α in saliva of patients with OSCC as compared to healthy individuals [20,52]. SahebJamee et al. also observed higher levels of IL-1α, IL-6, IL-8, and TNF-α in saliva of patients with OSCC in comparison with healthy subjects, however only differences in IL-6 concentration were statistically significant [53]. In turn, Punyani and Sathavane stated significantly higher salivary concentrations of IL-8 in OSCC patients in comparison with controls. The levels of IL-8 were also compared as per the TNM stage and histopathologic grading. The levels were the highest for stage IV disease, however, the difference between stages was statistically non-significant. The mean salivary IL-8 concentration was higher in patients with moderately differentiated squamous cell carcinoma than in patients with well-differentiated squamous cell carcinoma, however, the difference was also not statistically significant [45]. Korostoff et al. analyzed salivary levels of IL-1α, IL-6, IL-8, and TNF-α in patients with exophytic and endophytic tongue squamous cell carcinoma (TSCC) [54]. They observed an increasing trend of all assessed cytokines from controls to TSCC subjects. All cytokines were markedly elevated in saliva of patients with endophytic TSCC. Moreover, patients with endophytic TSCC and elevated IL-8 had a shorter lifespan after diagnosis.

In the present study levels of all analyzed cytokines were higher in saliva of patients with OPMDs with dysplasia than in subjects without oral mucosal lesions, however only differences in IL-8 concentrations were statistically significant (when only OSCC, OED, and healthy controls were taken into consideration). Rhodus et al. observed markedly higher salivary levels of IL-1α, IL-6, IL-8, and TNF-α in patients with oral lichen planus with dysplasia in comparison with the control group [20,53]. Sharma et al. reported markedly higher levels of IL-6 in patients with oral leukoplakia with dysplasia as compared to controls. Moreover, within the leukoplakia group IL-6 level was found to be increased with increase in the severity of dysplasia [55]. Lack of statistical significance in differences of IL-1α, IL-6, and TNF-α concentrations between patients with OED and controls in the present study could be related to the fact that all but only one case of OED were classified as mild, whereas in the study of Rhodus et al. all cases were classified as moderate or severe dysplasia. Similar to Rhodus et al., Kaur and Jacobs reported significantly higher levels of IL-6, IL-8, and TNF-α in saliva of patients with OPMDs (oral leukoplakia, oral lichen planus, and oral submucous fibrosis) in comparison with the control group [46]. They also observed that salivary levels of IL-6, IL-8, and TNF-α were markedly higher in the advanced stages of OPMDs as compared to the early stages. In turn, the study of Rajkumar et al. revealed significantly higher levels of IL-6 and TNF-α in patients with oral leukoplakia and oral submucous fibrosis in comparison with healthy individuals [52], whereas differences in IL-8 concentrations between patients with OPMDs and controls were not significant. Unfortunately, the authors of both mentioned studies did not give information about epithelial dysplasia of analyzed cases of OPMDs. In the study of Punyani and Sathavane, salivary levels of IL-8 were higher in patients with oral submucous fibrosis and oral leukoplakia in comparison with the controls, but the difference was statistically non-significant. It should be underlined that there was no histological evidence of dysplasia in all cases of oral submucous fibrosis and only in five of twelve cases of oral leukoplakia mild dysplasia was stated [45]. The results of the present study did not reveal significant differences in salivary concentration of IL-1α, IL-6, IL-8, and TNF-α between patients with OPMDs without dysplasia and healthy individuals.

In turn, we observed markedly higher levels of IL-8 in OSCC patients in comparison with OED cases. Rhodus et al. reported significantly higher levels of IL-1α, IL-6, IL-8, and TNF-α in OSCC patients as compared to patients with oral lichen planus with dysplasia, whereas Punyani and Sathavane stated markedly higher levels of IL-8 in the OSCC group in comparison with patients with oral leukoplakia, but only in five of twelve cases mild dysplasia were described [20,45,52]. Rajkumar et al. reported significantly higher levels of IL-6, IL-8, and TNF-α in patients with OSCC as compared to patients with oral leukoplakia and oral submucous fibrosis, however they gave no information about epithelial dysplasia [56].

There are two large scale studies in which diagnostic value of IL-8 and IL-6 for OSCC was assessed. Rajkumar et at. analyzed IL-8 levels in saliva of patients with OSCC and OPMDs (oral leukoplakia and oral submucous fibrosis). This study revealed significantly higher levels of IL-8 in patients with OSCC and OPMDs as compared to healthy individuals [57]. Moreover, they observed a significant increase in levels of salivary IL-8 in OSCC patients in comparison with OPMDs. Most cases of OPMDs were dysplastic. Receiver operating characteristic curve analysis found salivary IL-8 to have superior sensitivity in detecting OSCC. A significant increase in IL-8 levels based on the histologic grading of OSCC was also observed. Analogical results in case of IL-6 were reported by Dineshkumar et al. [58].

## 5. Conclusions

The present study confirmed that proinflammatory, NF-kappaB dependent cytokines are involved in pathogenesis of OPMDs and OSCC. The increase in salivary levels of IL-6, IL-8, and TNF-α could be a useful indicator of malignant transformation process within the oral mucosa. The higher levels of proinflammatory, NF-kappaB dependent cytokines, especially IL-8 and TNF-α in saliva of patients with OSCC or OPMDs, could be caused by increased expression of these cytokines in pathological tissues. The most important biomarker of malignant transformation process within oral mucosa among all assessed cytokines seems to be IL-8. It was present within all layers of the epithelium in most OSCCs, was not present in epithelial cells of specimens assessed as normal oral mucosa and was observed in the smallest number of OPMDs samples. Moreover, its salivary concentration was significantly higher in patients with OSCC as compared not only to healthy subjects but also to patients with OPMDs with dysplasia. Further studies on a large sample size are required to confirm the utility of proinflammatory, NF-kappaB dependent cytokines as screening/diagnostic markers for routine use at clinical practice.

## Figures and Tables

**Figure 1:**
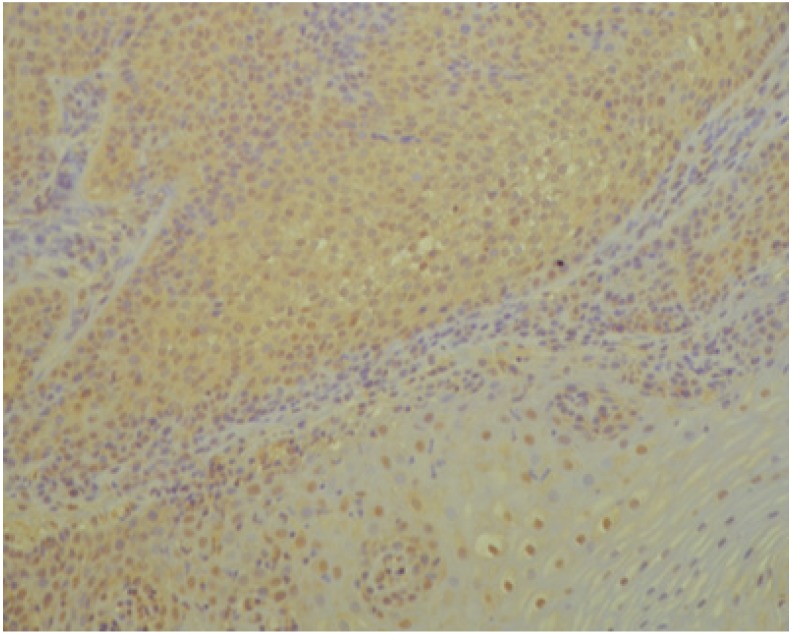
IL-1α—moderate brown staining in all epithelial cells of oral squamous cell carcinoma (OSCC) (20x).

**Figure 2:**
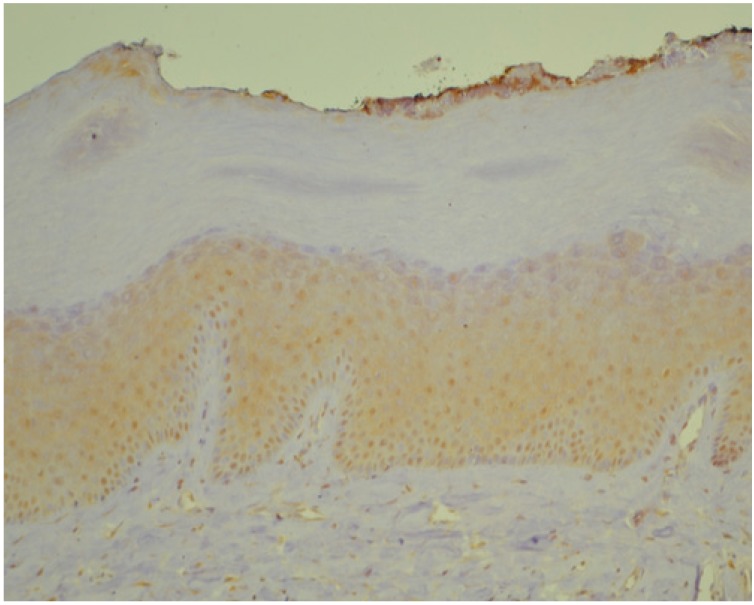
IL-1α—moderate brown staining in epithelum (all layers) of oral leukoplakia without dysplasia (OL) (20x).

**Figure 3:**
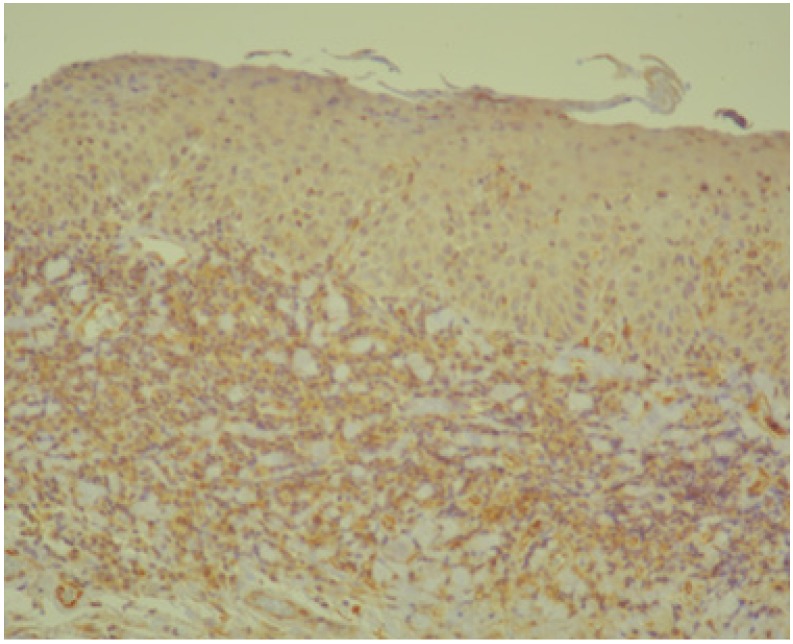
IL-6—moderate brown staining in epithelium (all layers) and strong staining in stroma of oral epithelial dysplasia (OED) (20x).

**Figure 4:**
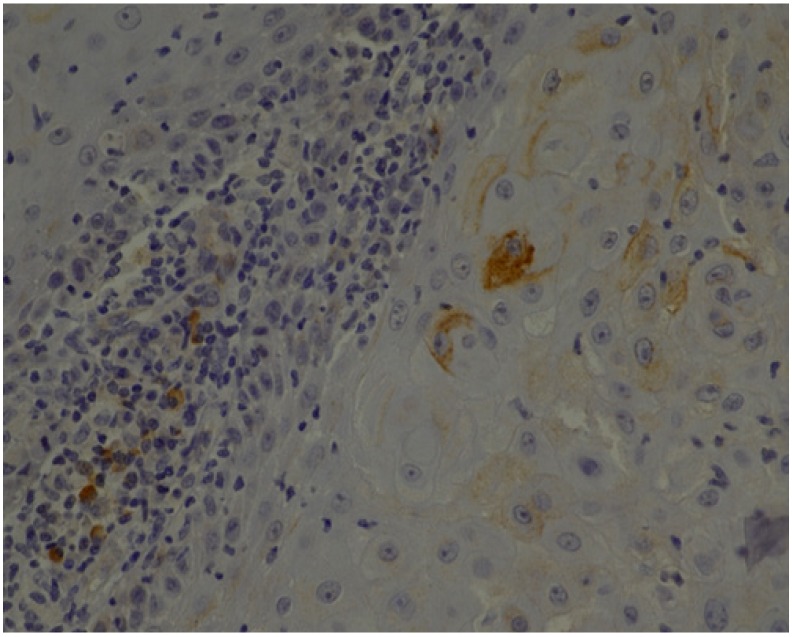
IL-8—moderate brown staining in epithelial cells and stroma of OSCC (40x).

**Figure 5:**
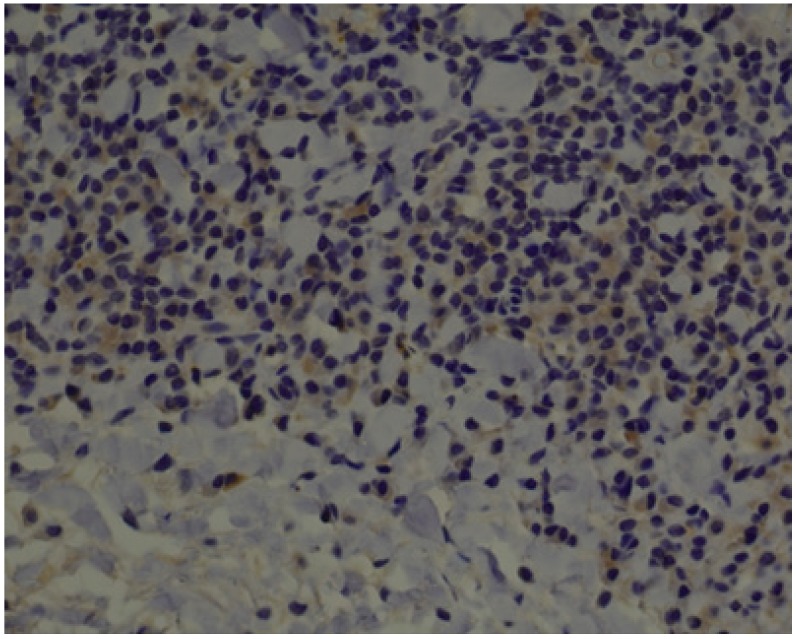
IL-8—weak brown staining in stroma of OED (40x).

**Figure 6:**
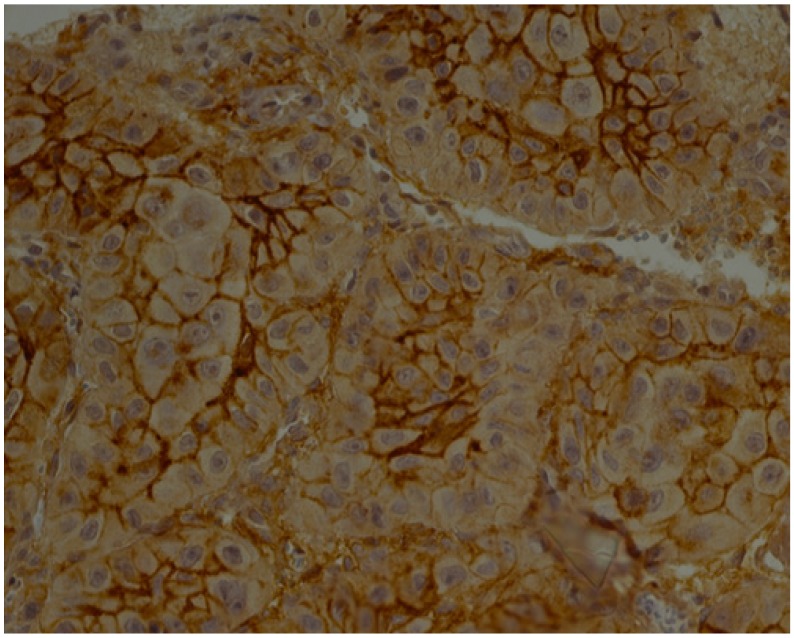
TNF-α—strong brown staining in epithelial cells of OSCC (40x).

**Figure 7:**
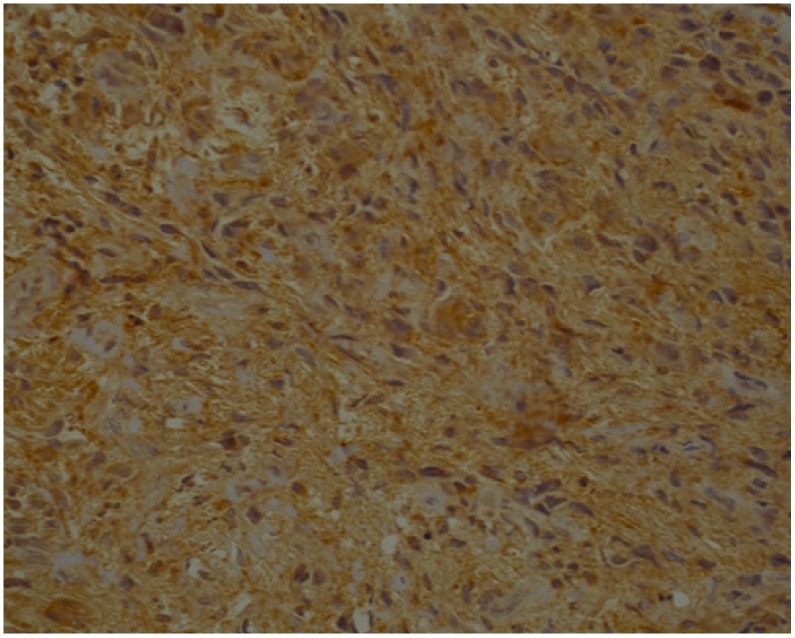
TNF-α—moderate brown staining in stroma of OSCC (40x).

**Figure 8:**
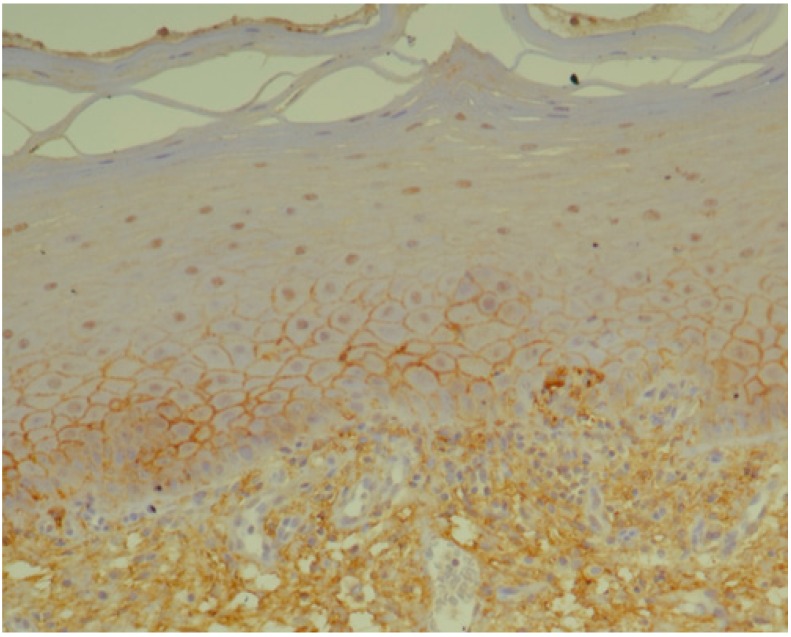
TNF-α—strong brown staining in epithelium (basal and parabasal layer) and stroma of oral lichen planus without dysplasia (OLP) (20x).

**Table 1:** Immunoreactive score for cytokines immunohistochemistry.

Score	Percentage of Immunopositive Cells	Staining Intensity
0	0	Negative
1	<5	Weak
2	5–50	Moderate
3	>50	Strong

All histopathological and immunohistochemical evaluations were made by board-certified specialist in pathomorphology (K.O.) in the Chair of Pathomorphology, Jagiellonian University Medical College, Krakow, Poland.

**Table 2:** Distribution of particular cytokines within the epithelium of specimens.

Interleukin	Distribution	NOM *n* = 7	OLP *n* = 15	OL *n* = 21	OED *n* = 10	OSCC *n* = 14	*p* *
IL-1⍺	none	2 (28.6)	3 (20.0)	5 (23.8)	1 (10.0)	1 (7.1)	0.011 (0.003 ′)
	basal	1 (14.3)	7 (46.7)	6 (28.6)	6 (60.0)	4 (28.6)	
	parabasal	4 (57.1)	4 (26.7)	4 (19.0)	3 (30.0)	1 (7.1)	
	all layers	0 (0.0)	1 (6.7)	6 (28.6)	0 (0.0)	8 (57.1)	
IL-6	none	2 (28.6)	3 (20.0)	2 (9.5)	3 (30.0)	4 (28.6)	0.117 (0.292 ′)
	basal	1 (14.3)	0 (0.0)	0 (0.0)	2 (20.0)	0 (0.0)	
	parabasal	2 (28.6)	1 (6.7)	3 (14.3)	0 (0.0)	1 (7.1)	
	all layers	2 (28.6)	11 (73.3)	16 (76.2)	5 (50.0)	9 (64.3)	
IL-8	none	7 (100.0)	7 (46.7)	13 (61.9)	6 (60.0)	3 (21.4)	0.101 (0.021 ′)
	basal	0 (0.0)	1 (6.7)	2 (9.5)	0 (0.0)	1 (7.1)	
	parabasal	0 (0.0)	3 (20.0)	2 (9.5)	1 (10.0)	1 (7.1)	
	all layers	0 (0.0)	4 (26.7)	4 (19.0)	3 (30.0)	9 (64.3)	
TNF-α	none	2 (28.6)	0 (0.0)	1 (4.8)	1 (10.0)	1 (7.1)	0.011 (0.005 ′)
	basal	0 (0.0)	0 (0.0)	0 (0.0)	0 (0.0)	1 (7.1)	
	parabasal	3 (42.9)	7 (46.7)	4 (19.0)	0 (0.0)	0 (0.0)	
	all layers	2 (28.6)	8 (53.3)	16 (76.2)	9 (90.0)	12 (85.7)	

NOM—normal oral mucosa, OLP—oral lichen planus without dysplasia, OL—oral leukoplakia without dysplasia, OED—oral epithelial dysplasia, OSCC—oral squamous cell carcinoma; * Fisher’s exact test; ′ *p*-value in case of comparison between three groups: OSCC, OED, and NOM.

**Table 3:** Immunoreactivity for particular cytokines in epithelium/cancer cells.

Immunoreactivity (Epithelium/Cancer Cells)	NOM *n* = 7	OLP *n* = 15	OL *n* = 21	OED *n* = 10	OSCC *n* = 14	*p* *
**IL-1α**						0.238 (0.169 ′)
Min	0.00	0.00	0.00	0.00	0.00	
Max	5.00	5.00	5.00	6.00	6.00	
Me	3.00	4.00	4.00	4.00	4.00	
IQR	5.00	5.00	5.00	6.00	6.00	
**IL-6**						0.176 (0.769 ′)
Min	0.00	0.00	0.00	0.00	0.00	
Max	5.00	5.00	6.00	5.00	6.00	
Me	4.00	4.00	5.00	3.00.5.00	4.00	
IQR	5.00	5.00	6.00	5.00	6.00	
**IL-8**						0.009 (0.006 ′)
Min	0.00	0.00	0.00	0.00	0.00	
Max	0.00	5.00	4.00	4.00	5.00	
Me	0.00	3.00	0.00	0.00	4.00	
IQR	0.00	5.00	4.00	4.00	5.00	
**TNF-α**						0.001 (0.003 ′)
Min	0.00	4.00	0.00	0.00	0.00	
Max	4.00	6.00	6.00	6.00	6.00	
Me	3.00	5.00	5.00	5.00	5.00	
IQR	4.00	2.00	6.00	6.00	6.00	

* Kruskal-Wallis test; ′ *p*-value in case of comparison between three groups: OSCC, OED, and NOM.

**Table 4:** Immunoreactivity for particular cytokines in stroma.

Immunoreactivity (Stroma)	NOM *n* = 7	OLP *n* =15	OL *n* = 21	OED *n* =1 0	OSCC *n* = 14	*p* *
**IL-1α**						0.064 (0.077 ′)
Min	3.00	3.00	4.00	4.00	3.00	
Max	5.00	6.00	6.00	6.00	6.00	
Me	4.00	5.00	6.00	5.00	5.00	
IQR	2.00	3.00	2.00	2.00	3.00	
**IL-6**						0.049 (0.150 ′)
Min	2.00	4.00	3.00	3.00	3.00	
Max	6.00	6.00	6.00	6.00	6.00	
Me	5.00	5.00	5.00	5.00	4.00	
IQR	4.00	2.00	3.00	3.00	3.00	
**IL-8**						0.012 (0.013 ′)
Min	0.00	2.00	2.00	2.00	2.00	
Max	3.00	5.00	5.00	4.00	5.00	
Me	2.00	3.00	3.00	3.00	3.00.5.00	
IQR	3.00	3.00	3.00	2.00	3.00	
**TNF-α**						0.001 (0.002′)
Min	2.00	4.00	4.00	5.00	4.00	
Max	5.00	6.00	6.00	6.00	6.00	
Me	5.00	6.00	5.00	6.00	5.00	
IQR	3.00	2.00	2.00	1.00	2.00	

* Kruskal-Wallis test; ′ *p*-value in case of comparison between three groups: OSCC, OED, and NOM.

**Table 5:** Study groups characteristics.

	CG		OLP		OL		OED		OSCC		*p*
	*n* = 9 (%)		*n* = 13 (%)		*n* = 16 (%)		*n*= 7 (%)		*n* = 9 (%)		
**Age**											0.56 *
Me (QR)	58.00 (29.00)		54.00 (37.00)		59.50 (43.00)		61.00 (31.00)		63.00 (49.00)		
M (SD)	58.11 (10.07)		56.00 (12.32)		60.75 (10.51)		66.14 (11.98)		61.00 (13.69)		
**Sex**											0.58 **
Male	4	(44.4)	6	(46.2)	6	(37.5)	2	(28.6)	6	(66.7)	
Female	5	(55.6)	7	(53.8)	10	(62.5)	5	(71.4)	3	(33.3)	
**Cigarette use**											0.6 **
Current	3	(33.3)	3	(23.1)	8	(50.0)	2	(28.6)	5	(55.6)	
Past	3	(33.3)	2	(15.4)	4	(25.0)	2	(28.6)	1	(11.1)	
Never	3	(33.3)	8	(61.5)	4	(25.0)	3	(42.9)	3	(33.3)	
**Alcohol use**											0.3 **
Regular	0	(0.0)	3	(23.1)	4	(25.0)	0	(0.0)	1	(11.1)	
Occasional	9	(100.0)	10	(76.9)	12	(75.0)	7	(100.0)	8	(88.9)	

CG—control group, OLP—patients with diagnosis of oral lichen planus without dysplasia, OL—patients with diagnosis of oral leukoplakia without dysplasia, OED—patients with diagnosis of oral epithelial dysplasia, OSCC—patients with diagnosis of oral squamous cell carcinoma; * Kruskal-Wallis test; ** Fisher’s exact test.
